# Supplementary material for: An Evaluation of the Performance of Five Burnout Screening Tools: A Multicentre Study in Anaesthesiology, Intensive Care, and Ancillary Staff
Source: J Clin Med. 2021 Oct 21;10(21):4836. doi: 10.3390/jcm10214836 (PMC8584380; doi:10.3390/jcm10214836)
Supplement: Supplementary file 1 [file jcm-10-04836-s001.zip › jcm-1376573-supplementary.pdf]

Supplementary Table S1. 36-item survey completed by respondents.

Survey Parts	Questions
Part 1 - MBI-HSS [a]<br>(22 items)	The MBI-HSS comprised of 22 proprietary items; SI-MBI and DI-MBI were derived from Question 8 and 10 of the MBI-HSS.
Part 2 - RBST (Figure 1) and SIBOQ[18]<br>(5 items)	SIBOQ:<br><br>"Please choose one of the following options that most accurately describes how you feel most of the time."<br><br>(1) I enjoy my work. I have no symptoms of burnout<br><br>(2) Occasionally I am under stress, and I don't always have as much energy as I once did, but I don't feel burned out<br><br>(3) I am definitely burning out and have one or more symptoms of burnout, such as physical and emotional exhaustion<br><br>(4) The symptoms of burnout that I'm experiencing won't go away. I think about work frustrations a lot<br><br>(5) I feel completely burned out and often wonder if I can go on. I am at the point where I may need some changes or may need to seek some sort of help.
Part 3<br>(9 items)	(1) What is your gender?<br><br>(2) What is your age?<br><br>(3) What is your role in the hospital?<br><br>(4) On average, how many hours do you work each week?<br><br>(5) Has the COVID-19 pandemic caused you more stress at work? (Yes/No)<br><br>(6) The COVID-19 pandemic has:<br>(a) reduced my workload, (b) not affected my workload,<br>(c) increased my workload<br><br>(7) Do you care for patients suspected with or have active COVID-19 infection? (Yes/No)<br><br>(8) What causes you the most amount of stress?<br>(a) My job, (b) My personal life, (c) Neither<br><br>(9) In your job, what causes you the most stress?

MBI-HSS = Maslach Burnout Inventory Human Services Survey, RBST = Rapid Burnout Screening Tool, SIBOQ = Single Item Burnout Question, COVID-19 = Coronavirus disease-2019

[a] Questions cannot be reproduced without a license (available from www.mindgarden.com).

Supplementary Table S2. Correlation between dimension-questions in burnout screening tools and average MBI-HSS dimension scores.

	**Emotional Exhaustion (EE)**	**Depersonalisation (DP)**	**Personal Accomplishment (PA)**
[a]MBI-Qn8: Spearman ϱ (95% CI)	0.86 (0.84 - 0.88)		
[a]MBI- Qn10: Spearman ϱ (95% CI)		0.77 (0.73 - 0.80)	
5Qn-EE aMBI: Spearman ϱ (95% CI)	0.96 (0.95 - 0.97)		
3Qn-DP aMBI: Spearman ϱ (95% CI)		0.93 (0.92 - 0.95)	
4Qn-PA aMBI: Spearman ϱ (95% CI)			0.95 (0.94 - 0.95)
RBST Qn1: Spearman ϱ (95% CI)	0.80 (0.77 - 0.83)		
RBST Qn2: Spearman ϱ (95% CI)		0.60 (0.53 - 0.64)	
RBST Qn3: Spearman ϱ (95% CI)			0.59 (0.53 - 0.65)
RBST Qn4: Spearman ϱ (95% CI)	0.78 (0.74 - 0.81)		
SIBOQ: Spearman ϱ (95% CI)	0.64 (0.59 - 0.69)		

MBI = Maslach Burnout Inventory (HSS), aMBI = 12-item abbreviated MBI, RBST = Rapid Burnout Screening Tool, SIBOQ = Single Item Burnout Question. Qn = question, 95% CI = 95% confidence interval. Grey cells indicate comparisons were not appropriate because of different burnout dimensions.

[a] Question 8 (Qn8) and Question 10 (Qn10) are featured in the single-item measures of burnout (SI-MBI) and the dual-item measure of burnout (DI-MBI). Both these tools will have identical Spearman's rho (ϱ).
